# Characterisation of the role of Vrp1 in cell fusion during the development of visceral muscle of *Drosophila melanogaster*

**DOI:** 10.1186/1471-213X-10-86

**Published:** 2010-08-11

**Authors:** Therese Eriksson, Gaurav Varshney, Pontus Aspenström, Ruth H Palmer

**Affiliations:** 1 Department of Molecular Biology, Building 6L, Umeå University, Umeå S-90187, Sweden; 2 Department of Microbiology, Tumor and Cell Biology (MTC), Karolinska Institute, Box 280, Nobels väg 16, SE-171 77 Stockholm, Sweden

## Abstract

**Background:**

In *Drosophila *muscle cell fusion takes place both during the formation of the somatic mesoderm and the visceral mesoderm, giving rise to the skeletal muscles and the gut musculature respectively. The core process of myoblast fusion is believed to be similar for both organs. The actin cytoskeleton regulator Verprolin acts by binding to WASP, which in turn binds to the Arp2/3 complex and thus activates actin polymerization. While Verprolin has been shown to be important for somatic muscle cell fusion, the function of this protein in visceral muscle fusion has not been determined.

**Results:**

Verprolin is specifically expressed in the fusion competent myoblasts of the visceral mesoderm, suggesting a role in visceral mesoderm fusion. We here describe a novel Verprolin mutant allele which displays subtle visceral mesoderm fusion defects in the form of mislocalization of the immunoglobulin superfamily molecule Duf/Kirre, which is required on the myoblast cell surface to facilitate attachment between cells that are about to fuse, indicating a function for Verprolin in visceral mesoderm fusion. We further show that Verprolin mutant cells are capable of both migrating and fusing and that the WASP-binding domain of Verprolin is required for rescue of the Verprolin mutant phenotype.

**Conclusions:**

Verprolin is expressed in the visceral mesoderm and plays a role in visceral muscle fusion as shown by mislocalization of Duf/Kirre in the *Verprolin *mutant, however it is not absolutely required for myoblast fusion in either the visceral or the somatic mesoderm.

## Background

In general there are three major muscle types in vertebrates as well as in insects; visceral muscle, cardiac muscle and skeletal muscle. *Drosophila *muscle progenitors, i.e. myoblasts, arise during embryogenesis and undergo the central process of myoblast fusion during the development of both the visceral and the somatic muscles. The mechanisms underlying cell fusion are actively studied in musculature of *Drosophila melanogaster*, with significant focus on the process of fusion within the somatic mesoderm (SM), although the phenomenon of myoblast fusion also occurs during the formation of the visceral muscle. The visceral mesoderm (VM) of the fruitfly consists of an inner layer of circular muscles, formed after one round of myoblast fusion, surrounded by an outer layer of longitudinal muscles [1-3]. Although the process of fusion in the VM is generally considered to be similar to SM fusion, VM fusion has not been as extensively studied and is not entirely understood [4-7]. To date, a number of molecules that are required for SM fusion have been identified, leading to the development of models describing the process of SM fusion [8]. Central to this, two different myoblast subtypes have been identified, founder cells (FCs) and fusion competent myoblasts (FCMs), which differentially express a number of transcription factors and adhesion molecules [9]. The FC is destined to become the first cell of each SM muscle, fusing with FCMs to generate the multinucleated muscle. FCMs continue to fuse with the growing myotube ultimately resulting in a muscle of the appropriate mass [10,11]. Attraction between the FC and the FCM is mediated, at least in part, by immunoglobulin-domain containing proteins such as protein Dumbfounded/Kin of Irre (Duf/Kirre) and Sticks and Stones (SNS) which are expressed on the cell membrane of the FCs and FCMs respectively [12-15]. The subsequent fusion of the myoblast plasma membrane is to a large extent dependent on signaling pathways regulating the actin cytoskeleton.

The significance of the actin machinery in SM fusion has become evident from studies of mutants of the Scar-Wasp signaling network. Scar (WAVE in mammals) and Wiskott-Aldrich syndrome protein (Wasp) are multidomain proteins which are structurally different at their NH_2 _-terminal domains, but which both contain a common Verprolin-homology, cofilin-homology, and highly acidic (VCA) - region at the COOH-terminal region, through which they bind to and activate the Arp2/3 complex [16]. The Arp2/3 complex is a well characterized actin nucleator, and thus Scar and Wasp are important regulators of actin polymerization [16]. A number of additional proteins are necessary for the proper function of both Scar and Wasp; Scar acts in a complex with four other proteins, including Kette (NAP125 in mammals), while Wasp functions in a complex with Verprolin (Vrp)[17]. Vrp is also known as Wasp interacting protein (WIP) in mammals [18] and in *Drosophila *Vrp is known as Verprolin1 (Vrp1) [19]/D-WIP [20]/Solitary [21]/and Solas [22]. Both Scar and Wasp are activated by small GTPases such as Rac and Cdc42 [23]. Rac, in turn, is regulated by the guanine nucleotide exchange factor Myoblast city (Mbc) [24]. *Drosophila *mutants in *Scar*, *Wasp*, *Vrp*, *Arp2*, *Kette*, *mbc*, *Rac1*, *Rac1*-*Rac2-mtl *and *Cdc42 *all show SM fusion defects during embryonic stages, although the severity of fusion phenotypes varies extensively between the different mutants, probably due to redundancy as well as maternal contribution in certain cases [20,21,25-28]. The fusion defects in these mutants, characterized by unfused SM cells as well as abnormal actin accumulations at the cell-cell attachment sites (in the case of Scar, Wasp, Kette, Rac1-Rac2-mtl), confirm the importance of the actin machinery in SM cell fusion [25,29].

In this work we have investigated the process of myoblast fusion in the VM. VM cells in *Drosophila melanogaster *express the ALK (Anaplastic lymphoma kinase) receptor tyrosine kinase (RTK), which activates a signaling cascade resulting in the specification of VM FCs [4-6]. The immunoglobulin-domain containing molecules Duf/Kirre and Sns are expressed in the VM FCs and FCMs respectively, and play a role in VM fusion, mediating adhesion between the FCs and FCMs. We identified the actin regulatory protein Vrp1 as a molecule important in the process of muscle fusion the SM and VM development, based on a deficiency screen for VM fusion mutants carried out in our laboratory. A role for Vrp in the SM fusion process has previously been reported [20-22,26], however, Vrp is also strongly expressed in the FCMs of the VM suggesting a role in VM fusion. Here we show that *Vrp1 *mutants display defects in the development of the visceral muscle, although the defects observed in the VM are more subtle than those observed in the SM.

## Results

### V*rp1*^*f06715 *^is an insertion in the *Vrp *locus which exhibits severe somatic muscle fusion defects

**Figure 1:**
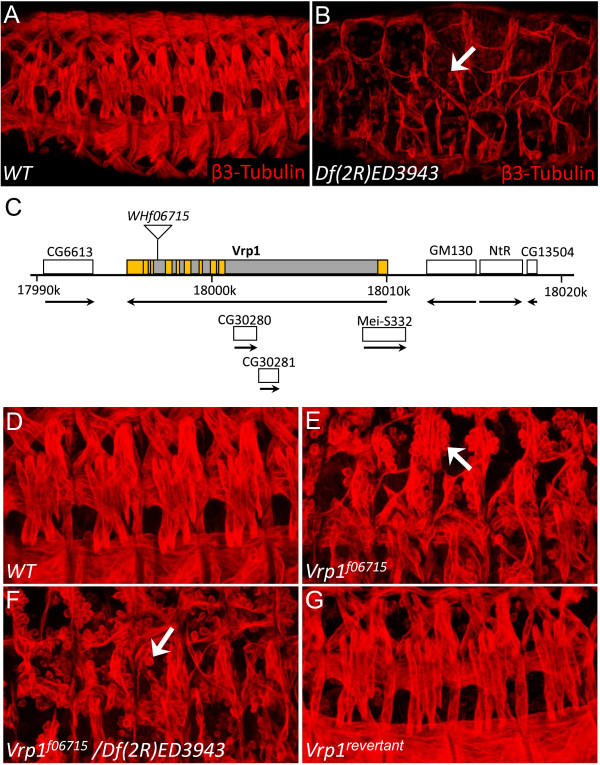
**V*rp1*^*f06715 *^is new *Vrp1 *allele which exhibits severe somatic muscle fusion defects**. (A-B, D-G) Stage 16 embryos were stained with antibodies against β3-Tubulin to visualize somatic muscles. (A) *Wild type *embryo (WT). (B) *Df(2R)ED3943 *mutant embryo with severe muscle fusion defects. Arrow indicates unfused cells. (C) Schematic representation of the *Vrp1*^*f06715 *^allele. The genomic location of the *Vrp1 *locus on 2R is indicated. mRNA representing exons and introns are shown as yellow and grey boxes respectively, and correspond to the longest predicted mRNA splice variant (CG13503-RA). The *Vrp1*^*f06715 *^allele has a piggyBac insertion (WHf06715) in the coding region of the 7^th ^intron, which disrupts gene function. Other genes in close proximity of the *Vrp1 *locus are illustrated with white boxes and their transcriptional direction with arrows (FlyBase [20]). (D) *Wild type *embryo (WT). (E) *Vrp1*^*f06715 *^mutant embryo (arrow indicates unfused cells). (F) *Vrp1*^*f06715*^/Df(2R)ED3943 transheterozygous embryo displaying the same muscle fusion defects as *Vrp1*^*f06715 *^(arrow indicates unfused cells). (G) The *Vrp1*^*f06715 *^phenotype was reverted by precise excision of the WHf06715 piggyBac element.

We initially identified the deficiency *Df(2R)ED3943 *as displaying a strong muscle fusion phenotype (Figure 1B). Examination of the genes contained within this region revealed the presence of *CG13503 *(originally named *Vrp1 *in Flybase), which had previously been identified as an FCM specific gene in the elegant microarray analysis of Estrada and Michelson 2008 [30]. Subsequent examination of the Harvard Exelixis stock collection revealed the presence of a potential *Vrp1 *mutant fly strain. This fly strain contained a piggyBac element inserted within the coding region of the *Vrp1 *gene (Figure 1C) and was therefore named *vrp1*^*f06715*^. This insertion completely disrupts *Vrp1 *gene function and causes lethality and a severe somatic muscle phenotype at the embryonic stage, both alone (Figure 1E) and in combination with *Df(2R)ED3943 *(Figure 1F). *Vrp1*^*f06715 *^embryos display a similar degree of somatic muscle fusion phenotype as the previously published *Vrp1 *mutant *D-WIP*^*D30 *^[21] [Additional file 1: Supplemental Figure 1], and *Vrp1*^*f06715*^*/D-WIP*^*D30 *^transheterozygotes [Additional file 1: Supplemental Figure 1], confirming *Vrp1*^*f06715 *^as a novel *Vrp1 *mutant allele. In addition, expression of the piggyBac transposase in *Vrp1*^*f06715 *^flies resulted in the mobilization and excision of *Vrp1*^*f06715 *^and reversion of the muscle fusion phenotype (Figure 1G), demonstrating that the *Vrp1*^*f06715 *^insertion indeed causes the fusion phenotype.

### Vrp expression pattern

**Figure 2:**
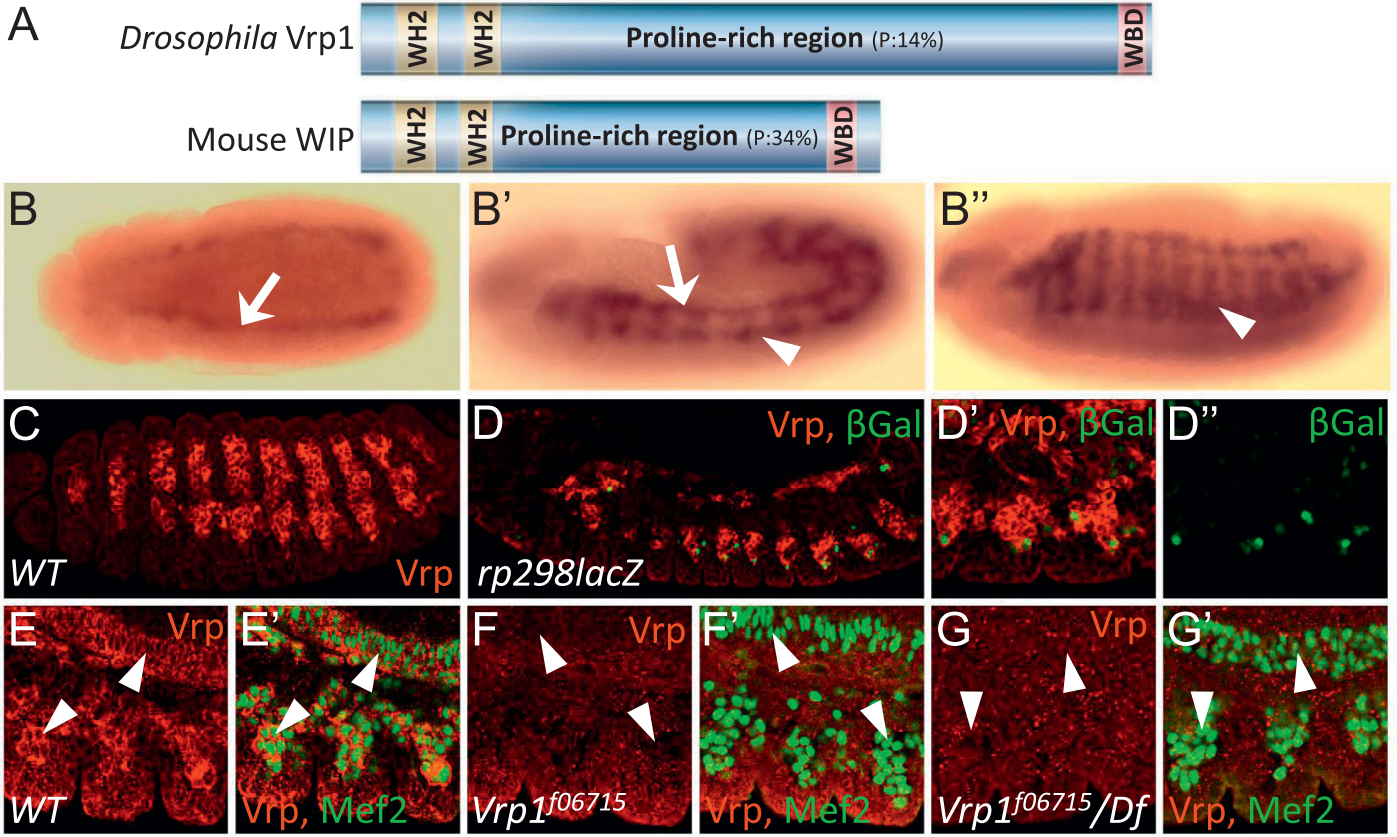
***Vrp1 *domain organization and expression pattern**. (A) Domain organization of Vrp1 proteins from *Drosophila *and mouse. Vrp1 has two WH2 domains (yellow boxes) and a WASP binding domain (pink box). Vrp1 is a very proline rich protein and the proline content is indicated in the Figure. (B-C) Vrp1 mRNA and protein expression patterns. (B) RNA *in situ *hybridization with *Vrp1 *anti-sense mRNA on *wild type *embryos. *Vrp1 *mRNA expression is detected in the visceral mesoderm at stage 11, dorsal view (arrow). (B'') At Stage 12 *Vrp1 *mRNA is visible in the somatic mesoderm (arrowhead) and visceral mesoderm (arrow), lateral view. (B''') *Vrp1 *mRNA expression in the somatic mesoderm of a stage 14 embryo (arrowhead), lateral view. (C) Vrp1 protein is detected with anti-Vrp1 antibodies in muscles of *wild type *embryos. (D) *Rp298lacZ *embryo, stained with anti-Vrp1 and anti-βGal antibodies. LacZ expression is detected in a FC cell specific pattern reflecting *Duf/Kirre *gene expression. No overlap between Vrp1 and β-gal expression is observed at early stages, indicating that Vrp1 is expressed only in the FCMs. (D') Close up of an *rp298lacZ *embryo, Vrp1 and β-gal expression. (D'') Close up of *rp298lacZ *embryo, β-gal expression alone. (E-G) Specificity of the Vrp1-antibodies is shown. (E) In *wild type *(WT) embryos Vrp1-antibodies detect Vrp1 protein in both SM and VM cells, indicated by arrowheads. (E') Nuclear Mef2 expression is detected in Vrp1 expressing cells (arrowheads). (F and G) No Vrp1 protein is detected in *Vrp1*^*f06715 *^embryos (compare F with E) or in *Vrp1*^*f06715*^*/Df(2R)ED3943 *transheterozygous embryos (compare G with E) by the Vrp1 antibodies (arrowheads indicate absence of Vrp1 expression). (F' and G') Mef2 expression indicates the position of VM and SM cells (arrowheads indicate absence of Vrp1 expression in Mef2 expressing cells).

The Vrp protein domain structure has been conserved throughout evolution from yeast to *Drosophila *and further on to higher organisms such as mouse and human. Vrp is a proline rich protein with two WH2 domains in the N-terminal region of the protein and a WASP-binding domain in the C-terminal portion (Figure 2A). *Vrp1 *mRNA is expressed in both the developing visceral (Figure 2B, arrows) and somatic muscles (Figure 2B, arrowheads). Anti-Vrp1 antibodies were generated in order to analyze the expression of the Vrp1 protein. Vrp1 protein is strongly expressed in muscles (Figure 2C and 2E) and is not detectable in *Vrp1*^*f06715 *^(Figure 2F) or in *Vrp1*^*f06715*^/Df(2R)ED3943 embryos (Figure 2G). Analysis of *rp298lacZ *embryos, which express beta galactosidase (lacZ) in the founder cell specific pattern of the *Duf/Kirre *gene[13,31], indicates that Vrp expression is specific for FCMs, since no overlap between Vrp and lacZ expression was detected (Figure 2D).

### Vrp is specifically expressed in the FCMs of the VM

**Figure 3:**
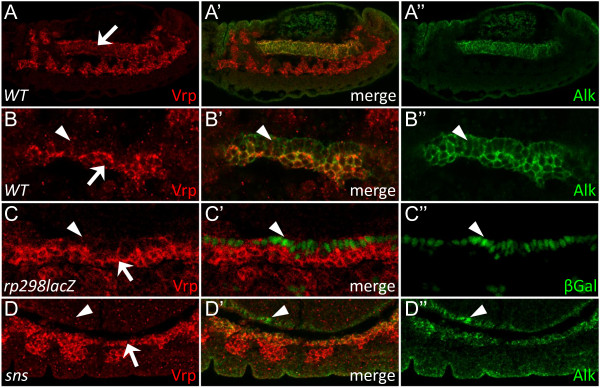
**Vrp1 is specifically expressed in the FCMs of the VM**. (A-B) *Wild type *embryos were stained for Vrp1 (red) and Alk (green) to visualize the VM. (A) Stage 14 embryo, lateral view. Vrp1 protein is expressed in the Alk positive VM (arrow indicates VM). (B) VM of a stage 11 embryo just after FC specification. Vrp1 protein expression is only detected in the pebble shaped FCMs (arrow) and not in the columnar FCs (arrowhead). (C) *rp298lacZ *stage 11 embryo stained for Vrp1 (red) and LacZ (green). Vrp1 is specifically expressed in the FCMs (arrow) and absent in the FCs (arrowhead). (D) *Sns *mutant stage 14 embryo stained for Vrp1 (red) and Alk (green). Vrp1 protein is expressed only in the FCMs (arrow) which have separated from the FCs (arrowhead).

An essential role for Vrp1 in somatic muscle fusion has been elegantly described in previous work [20-22], however its role in other tissues has not been studied. We observed that both Vrp1 mRNA and protein are found not only in the somatic muscles but also in the visceral mesoderm (Figure 2B arrow, and Figure 3A, arrow) as well as at muscle attachment sites (data not shown). The VM forms the midgut in the fruitfly, and at early embryonic stages, prior to fusion, columnar shaped FCs and the rounder FCMs of the VM can be distinguished morphologically as described previously [1,3]. Analysis of the VM of control embryos revealed expression of Vrp1 specifically in the FCMs (Figure 3B, arrow), while the columnar FCs lack Vrp1 expression (Figure 3B; arrowhead). Both FCMs and FCs express Alk, which outlines all VM cells (Figure 3A'', B'', D''). Vrp1 protein localization was further examined in *rp298lacZ *embryos, in which the FCs express lacZ [13,31], confirming the specificity of Vrp1 expression in FCMs (Figure 3C, arrow indicates FCMs, arrowhead indicates FCs). The specific expression of Vrp1 in FCMs can clearly be observed in *sns *mutant embryos, in which the FCs and the FCMs of the VM separate as a result of defective adhesion between the FCs and FCMs (Figure 3D, arrow indicates FCMs, arrowhead indicates FCs)[3,32]. Taken together, these results clearly demonstrate that Vrp1 is a FCM specific protein in the developing visceral mesoderm.

### The VM of Vrp1 mutant embryos displays a subtle phenotype

**Figure 4:**
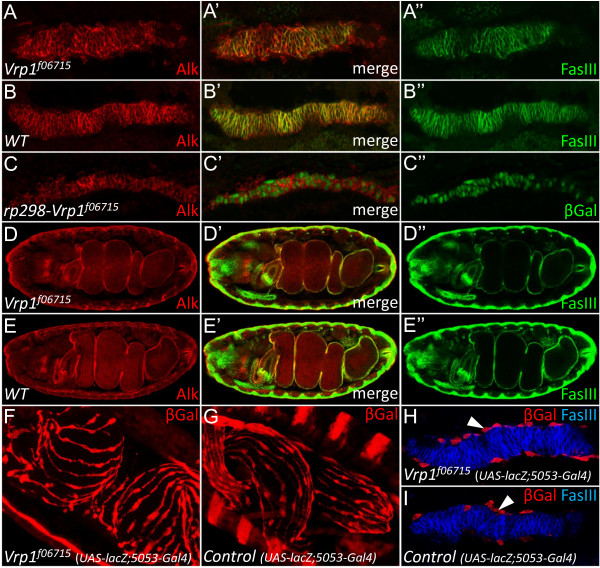
**The VM of *Vrp1 *mutant embryos displays subtle phenotypes**. (A-E) Embryos were stained for Alk to visualize the VM and Fasciclin III (FasIII) to indicate differentiated VM cells. (A) The VM of a *Vrp1*^*f06715 *^embryo is slightly unorganized but has no obvious fusion defects as cells fuse and form a gut structure later in development. (B) *Wild type *control. (C) VM of a *rp298;Vrp1*^*f06715 *^embryo, which expresses LacZ in the FC specific pattern of the *Duf/Kirre *gene. All cells of the VM express LacZ, indicating that FCs and FCMs have fused. (D) Late stage *Vrp1*^*f06715 *^embryo exhibit a wild type gut (compare with E). (E) Late stage control embryo. (F-I) Longitudinal muscles of the VM develop normally in *vrp1*^*f06715 *^mutants. LacZ is expressed in the longitudinal muscles of *Vrp1*^*f06715 *^mutants and control embryos using the5053-Gal4 driver. Anti-βGal staining (red) marks the longitudinal muscles. (F) *Vrp1*^*f06715 *^mutant embryo (*Vrp1-UAS:lacZ;5053-Gal4*), stage 17, shows a longitudinal muscle pattern similar to heterozygous controls (compare with G). (G) Heterozygous control embryo (*Vrp1-UAS:lacZ;5053-Gal4/CyOLacZ*), stage 17. In heterozygous animals βGal stains both longitudinal muscles and the striped pattern of the *wingless-LacZ *balancer chromosome. (H) Stage 12 *Vrp1*^*f06715 *^embryo (homozygous *Vrp1-UAS:lacZ;5053-Gal4*), exhibits longitudinal muscles with no obvious defect. FasIII (blue) marks the circular VM (arrowhead indicates longitudinal muscles). (I) Stage 12 heterozygous control embryo (*Vrp1-UAS:lacZ;5053-Gal4/CyOLacZ*). Longitudinal muscles (arrowhead) surround the FasIII expressing circular muscle.

While the expression of Vrp1 in the VM is confined to the FCM subtype as in the developing somatic muscle, the role of Vrp1 in VM muscle fusion does not seem to be as profound as in the SM. In the VM of *Vrp1*^*f06715 *^embryos the FCs and the FCMs appear to fuse despite the absence of Vrp1 protein (Figure 4A). The VM of the *Vrp1*^*f06715 *^mutants appears slightly disorganized at early stages (Figure 4A), however the development of the gut proceeds, and the *Vrp1*^*f06715 *^mutants develop a gut structure with midgut constrictions at later stages (Figure 4D). Examination of *Duf/Kirre *expression in the VM of stage 13 *Vrp1*^*f06715 *^embryos employing the *rp298lacZ *reporter [13,31] indicates that all VM myoblasts have fused as all cells appear to express LacZ (Figure 4C).

We also investigated the development of the longitudinal visceral muscles in *vrp1*^*f06715 *^mutants, employing UAS-LacZ expressed under the control of 5053-GAL4 as a readout. At stage 12 in both *vrp1*^*f06715 *^and control embryos (Figure 4H and 4I) the longitudinal muscles surround the circular musculature, and at later stages, both in mutants and controls, the longitudinal muscles form a characteristic longitudinal pattern (Figure 4F and 4G). These results indicate that longitudinal muscle development is not obviously affected by *Vrp1 *mutation.

**Figure 5:**
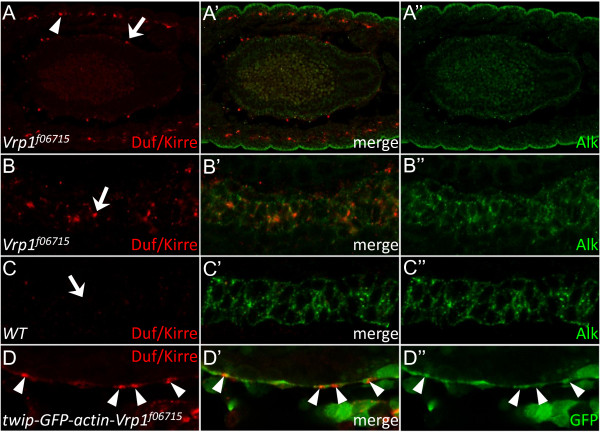
**Duf/Kirre is mislocalized in *Vrp1*^*f06715 *^mutants**. (A-C) Embryos stained for Duf/Kirre (red) and Alk (green). (A) Duf/Kirre accumulates in punctate foci in *Vrp1*^*f06715 *^mutant embryos (arrow). No Duf/Kirre expression can be detected at this stage in controls (see C). (B) Close up of VM in a *Vrp1*^*f06715 *^embryo in which accumulation of Duf/Kirre can be detected (arrow). (C) In *wild type *embryos Duf/Kirre expression is low and is not visible (arrow indicates absence of Duf/Kirre accumulations). (D) *twip-GFP-actin-Vrp1*^*f06715 *^embryo, in which a GFP-actin fusion protein is expressed under the control of the twist promoter [25]. After VM fusion Duf/Kirre accumulation in the VM is visible (arrowheads), but these show no obvious build-up of actin-GFP, indicating that the Duf/Kirre accumulations do not contain elevated actin expression.

To further examine the VM development in *Vrp1*^*f06715 *^mutant embryos we investigated a number of molecules which are known to play a role in myoblast fusion and muscle development. One such molecule; Duf/Kirre, is known to play a role in muscle cell fusion. Interestingly, we observed that Duf/Kirre protein is inappropriately expressed in muscles of *Vrp1*^*f06715 *^mutant embryos. During the course of this work we have observed that Duf/Kirre protein is normally highly expressed in the VM during stage 11, but after muscle cell fusion has occurred Duf/Kirre is downregulated and protein expression is undetectable after fusion, in keeping with a previous report from Menon *et. al. *[33] in the SM. In contrast, Duf/Kirre is observed in a punctuate pattern and appears not to be downregulated correctly in either the VM or SM of later stage *Vrp1*^*f06715 *^mutant embryos (Figure 5A and 5B, arrows, compared to wild type embryo Figure 5C, arrow), a phenomenon also observed in several SM fusion mutants [33]. One explanation is that these Duf/Kirre rich accumulations arise between VM FCs and FCMs when fusion does not proceed as normal.

Since actin foci have been reported to be formed at the cell-cell attachment sites between fusing FCs and FCMs, and to contain fusion proteins such as Sns, Rols, Loner, Blow and Mbc [25], we investigated if the Duf/Kirre accumulations in the *Vrp1*^*f06715 *^mutant could involve such actin structures. For this we employed the *twip-GFP-actin *fly strain in which a GFP-actin fusion protein is expressed under the control of the *twist *promoter [25], and examined actin localization in *Vrp1*^*f06715 *^mutant animals. Analysis of these mutants revealed that the Duf/Kirre accumulations do not contain elevated levels of actin (Figure 5D arrowheads), suggesting that the Duf/Kirre containing structures we observe are different than the above described actin foci.

### Mutations in additional components of the Scar-Wasp signaling network display similar phenotypes as *Vrp1*^*f06715*^

**Figure 6:**
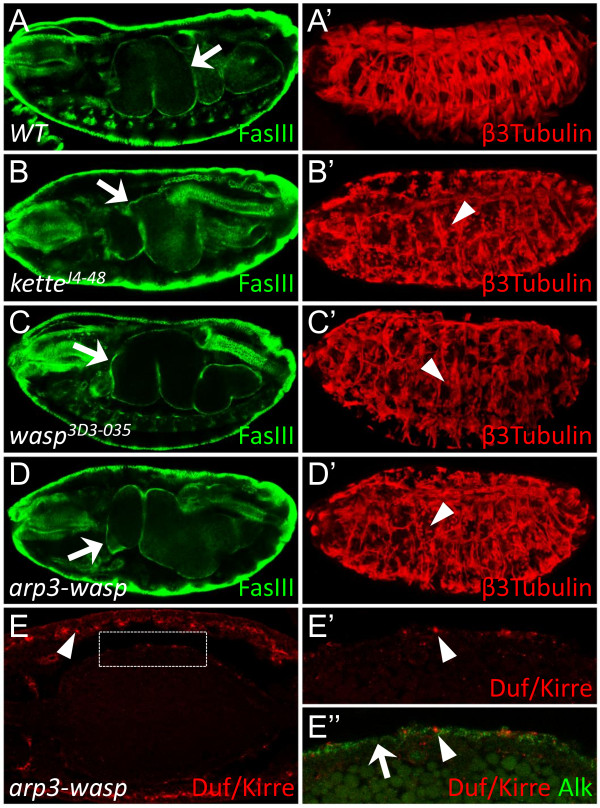
**Several mutants for components of the Scar-Wasp signaling network develop a normal gut, but display Duf/Kirre accumulation phenotype**. (A-D) Stage 17 embryos stained with FasIII to visualize VM and β3-Tubulin to visualize SM. All mutants in B-D display no obvious VM phenotypes, shown by the presence of a developed gut (arrows), this is despite severe SM mutant phenotypes (arrowheads indicate unfused SM cells in B'-D'). (A-A') *Wild type*. (B-B') *kette*^*J4-48*^. (C-C') *wasp*^*3D3-035*^. (D-D') *arp3-wasp*. (E) *arp3-wasp *mutant embryo, stage 15, stained with Duf/Kirre (red) and Alk (green). Duf/Kirre accumulations are observed in both the VM (box) and SM (arrowhead). (E') Close up of box in D. Duf/Kirre is accumulated in foci indicated by arrowheads. (E'') Close up of box in D, Alk staining marks VM (arrow). Arrowhead indicates Duf/Kirre accumulations as in H'.

Because the development of the VM appears to be less sensitive to perturbations in the actin regulating machinery than the SM, we decided to investigate the consequence of manipulating additional actin regulating proteins in the VM. We examined three mutants for components of the scar-wasp signaling network; *kette*^*J4-48*^, *wasp*^*3D3-035*^, and *arp3-wasp*. *Kette*^*J4-48 *^is a null mutant for the *kette *gene [27,34], *wasp*^*3D3-035 *^is a mutant allele that encodes a dominant negative form of Wasp and thereby also inhibits maternally contributed Wasp protein [35], and *arp3-wasp *is a double mutant of *wasp*^*3D3-035 *^and *arp3 *(which the latter encodes a component of the Arp2/3 complex [26]). We find that all three mutants; *kette*^*J4-48*^, *wasp*^*3D3-035*^, and *arp3-wasp*, exhibit normal VM development, resulting in the formation of a gut (Figure 6B - D, arrows indicate gut, and Figure 6D'', arrow indicates VM) despite severe SM fusion phenotypes (Figure 6B '- D', arrowheads indicate unfused SM cells). The Duf/Kirre rich accumulations observed in the *Vrp1*^*f06715 *^mutant were found to be present in all mutants examined (Figure 6E, and data not shown). Taken together, these data suggest that Arp2/3 mediated actin polymerization is not essential for the formation of the embryonic VM, in contrast to its indispensable role in the somatic muscle fusion process. It is possible that complete fusion is not an essential process in embryonic VM formation, and that therefore disrupting fusion mechanisms does not affect VM development significantly. However, the presence of Duf/Kirre accumulations indicates that some as yet uncharacterised defect in development of the VM exists.

### *Vrp1 *mutant VM cells are capable of migrating and fusing

In addition to having a role in muscle cell fusion, Vrp1 and other actin regulating proteins have, in other experimental systems, been suggested to have roles in cell motility [36-39]. In order to test the role of Vrp1 in both muscle fusion and cell motility experimentally we analyzed *Alk *mutant embryos. In *Alk *mutants, it has previously been shown that FCMs of the VM are able to migrate towards and fuse with the somatic muscle cell population [4-6].

**Figure 7:**
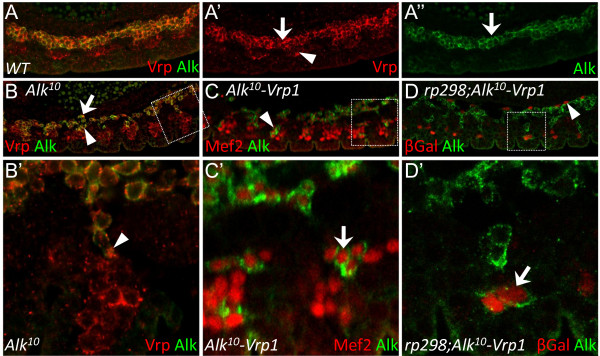
***Vrp1 *mutant cells of the VM are capable of migrating and fusing**. (A) The VM of a stage 12 *wild type *embryo stained with antibodies against Vrp (red) and Alk (green). At this stage FCs and FCMs have just fused. (A') Vrp protein expression is detected in VM cells (arrow) and SM cells (arrowhead). (A'') Alk protein expression is detected in VM only (arrow). (B-D) Examination of *Alk *mutant cells migrating from the VM to the SM in stage 12 embryos. (B) *Alk*^*10 *^mutant embryo, where unfused FCMs of the VM, which express Alk and Vrp, migrate towards the SM to fuse with the somatic FCs. SM cells are identified by Vrp protein expression and lack of Alk expression (arrow indicates unfused, detached VM cells and arrowhead indicates VM cells that have migrated into the SM). (B') Close up of B, showing an Alk and Vrp positive cell from the VM stretching out toward a Vrp1 positive cell of the SM (arrowhead indicates Vrp1 expression at the protrusion of the stretched VM cell). (C) *Alk-Vrp1*^*f06715 *^double mutant embryo displays a similar phenotype to that observed in the *Alk *single mutant where Alk expressing VM cells are detected among Mef2 expressing SM cells (arrowhead). (C') Close up of C shows Alk positive VM cells which have migrated into the SM cell population (arrow), hence *Vrp1 *disruption does not appear to affect the migration process of VM cells *in vivo*. (D) The VM of a *rp298;Alk-Vrp1 *embryo confirms that *Alk-Vrp *double mutant cells from the VM can fuse with FCs of the SM. Only FCs of the SM (and longitudinal muscles, arrowhead) express lacZ under the *Duf/Kirre *promoter in an *Alk *mutant embryo, hence cells that expresses both lacZ and Alk are FCMs originating from the VM which have fused with a FC of the SM (arrow).

In *wild type *stage 12 embryos Alk is expressed in the VM (Figure 7A'', arrow), while Vrp1 is expressed both in the VM (Figure 7A', arrow) and the SM (Figure 7A', arrowhead). In *Alk10 *mutant embryos at stage 12 there is no fusion of the VM and a number of mutant Alk expressing VM myoblasts have migrated to the SM (Figure 7B, arrowhead). Vrp1 protein can be detected in the leading tip of cells stretching towards a SM cell (Figure 7B', arrowhead), suggesting a possible role for Vrp1 in the migration mechanism. However, we observe that loss of Vrp1 has no appreciable effect on the migration of *Alk *mutant myoblasts of the VM, since Alk-positive cells can be detected in the somatic muscle cell populations of *Alk-Vrp1 *double mutants with a similar efficiency to that of *Alk *mutants. (Figure 7C, arrowhead, and 7C', arrow). To further investigate whether *Alk-Vrp1 *mutant myoblasts are capable of fusing with somatic FCs, we examined *Alk-Vrp1 *double mutant embryos carrying the *rp298lacZ *enhancer trap, which marks the FC population [13,31]. In these embryos, Alk positive lacZ expressing cells could readily be detected suggesting that *Alk-Vrp1 *mutant cells of the VM are able to both migrate and fuse with cells of the SM (Figure 7D and 7D', arrow). The fact that Vrp1 mutant cells are capable of fusing suggests that the fusion defects observed in the somatic muscles of *Vrp1 *mutant embryos are not caused by a complete block in fusion, but may reflect an inability of *Vrp1 *mutant cells to either complete the fusion process or to go through multiple rounds of fusion.

### Expression of Vrp1 in the FCM population rescues fusion

**Figure 8:**
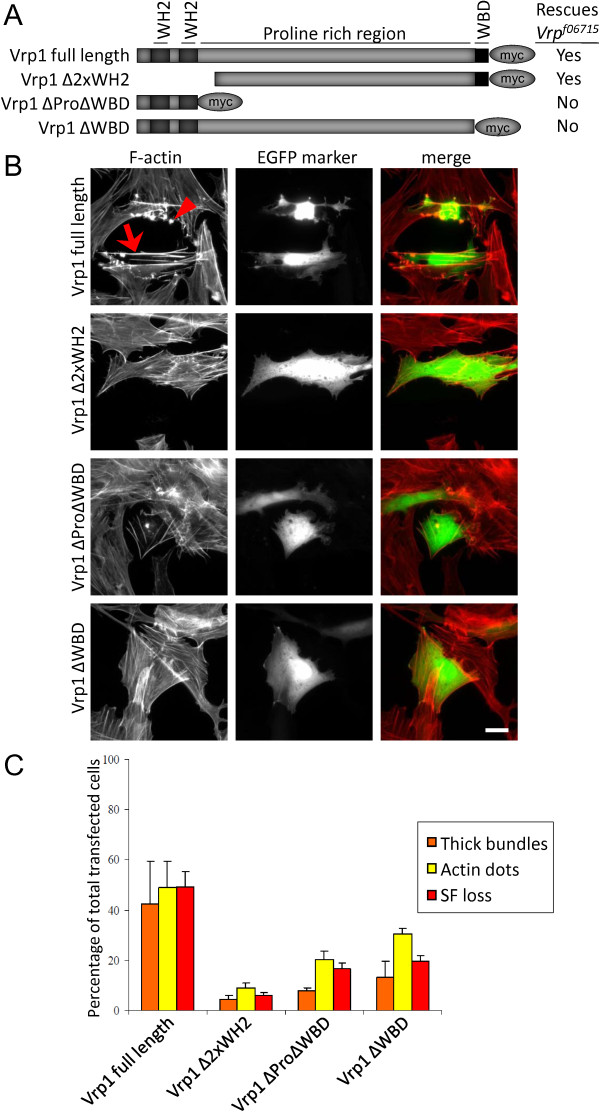
**Expression of Vrp1 in the FCM population rescues fusion and lethality in *Vrp1*^*f06715 *^mutants**. (A) Overview of the transgenic constructs generated for UAS-Gal4 fly experiments and for cell culture overexpression experiments. Dark grey boxes represent the WH2 domains, light grey box denotes the proline rich domain (Pro), black box is the WASP-binding domain (WBD). Myc-tag is indicated by an oval. Various domains of the Vrp1 protein were deleted as shown. Transgenes containing the Wasp-binding domain were able to rescue the *Vrp1*^*f06715*^mutant lethality when specifically expressed in the FCMs using a sns-GAL4 driver, while those transgenes lacking the Wasp-binding domains were unable to rescue the lethality of the *Vrp1*^*f06715*^mutant as indicated in the table. (B) Ectopic expression of the full length Vrp1 transgene, but not the truncated forms, induced a dramatic reorganization of the actin filament system in form of the assembly of thick bundles and the formation of actin dots, resulting in loss of stress fibers. Actin dots (accumulation of actin in foci, red arrowhead) and thick bundles (thick actin filaments, red arrow) are known to be formed upon ectopic expression of actin reorganizing proteins, such as mammalian Vrp1, at the expense of the stress fibers. A detailed description of the phenotypes are given in [Additional file 2: Supplemental Figure 2E]. Filamentous actin was visualized by TRITC-labeled phalloidin (red). Vrp1-expressing cells were detected by co-transfecting an EGFP- and Vrp1-expressing plasmids. Bar represents 20 μm. (C) Quantification of the effects on the actin organization caused by ectopic expression of the Vrp1 transgenes in PAE cells was performed; the percentage of cells displaying extensive stress fiber loss, thick bundles and actin dots were counted manually employing a 63x immersion oil objective. The values represent triplicates of analyzes of at least 100 transfected cells.

The Vrp1 protein contains a number of functional domains; two WH2 domains at the NH_2 _-terminal region, which are predicted to be actin binding domains, a central proline rich region, which are often involved in multi-protein complex formation, and at the COOH-terminal a WASP-binding domain, which facilitates binding to WASP [18,40]. In order to investigate the importance of the various domains of Vrp1 functionally we generated a set of transgenic *Drosophila *carrying UAS-Vrp1 transgenes (shown schematically in Figure 8A). In embryos employing either the Twist-Gal4 or Sns-Gal4 driver lines to ectopically express the various Vrp1 proteins we were unable to observe any visible phenotypes in the VM, nor with overexpression of the same proteins in imaginal discs (data not shown). In rescue experiments we found that both the full length Vrp1 transgene and the Vrp1^ΔWH2 ^proteins were able to fully rescue the *Vrp1 *mutant phenotype when overexpressed specifically in FCMs of *Vrp1 *mutants using the sns-Gal4 driver [41] (Figure 8A), as well as with the stronger muscle specific driver TwistGal4 (data not shown). In contrast, those transgenes which lacked the WASP-binding domains; Vrp1^ΔProΔWASP ^and Vrp1^ΔWASP^, were both unable to rescue either lethality (Figure 8A) or the somatic muscle phenotype to any extent using either of the two drivers [Additional file 2: Supplemental Figure 2B-D].

In parallel, we examined the effect of the various Vrp1 proteins on the organization of the actin cytoskeleton in porcine aortic endothelial (PAE) cells, reasoning that in this system we would be able to analyze the effect of the various Vrp1 protein domains on the morphology of the actin cytoskeleton. We have previously found that ectopic expression of mammalian Verprolin results in a profound reorganization of filamentous actin [42]. We observe a shift in the balance between monomeric and filamentous actin, seen as the bundling of stress fibers into thick actin filaments and the formation of actin foci (Figure 8B). Here, the full length Vrp1 transgene, but not the truncated forms, induced thick bundles, actin dots and stress fiber loss (Figure 8B and quantification in C), indicating that ectopic expression of Vrp1 regulates the organization of the actin cytoskeleton in PAE cells, in a similar manner to the mammalian Verprolins WIRE and WIP [42][Additional file2: Supplemental Figure 2E].

## Discussion and Conclusions

*Df(2R)ED3943 *was identified in a deficiency screen designed to identify novel genes with roles in VM development. Subsequent work led to the identification of the *Vrp1*^*WHF06715 *^mutant allele, present in the Exelixis mutant collection maintained at Harvard [43], which carries a piggyBac insertion in the *Vrp1 *gene. Closer examination of both *Df(2R)ED3943 *and the *Vrp1*^*f06715 *^mutant, lead to the identification of a subtle VM-phenotype as well as a severe somatic mesoderm (SM) fusion phenotype. At this time the SM fusion phenotype of independent mutants in the *Vrp1 *locus, which is characterized by a large number of unfused myoblasts, was unpublished. However, several elegant studies have subsequently described the role of Vrp1/D-WIP/Solitary/solas [20-22]. Therefore, we have focused upon investigation of the role of Vrp1 in the development of the visceral musculature.

The VM phenotype observed in *Vrp1*^*f06715 *^mutants is not as explicit as that in the SM. Both *Df(2R)ED3943 *and *Vrp1*^*f06715 *^exhibit defects in gut structure, however, we cannot definitively address how much of this is due to the lack of structural support of a surrounding somatic musculature. More detailed analysis of the developing VM of *Vrp1*^*f06715 *^mutant embryos was performed, leading to the discovery of a VM phenotype characterized by mislocalization of the adhesion molecule Duf/Kirre (see below for further discussion).

To date, there are few published mutants with strong VM fusion phenotypes, and even mutants with a complete block of fusion between myoblasts in both the SM and the VM, such as *sns *[1] and *myoblast city *[1,3] mutants, display subtle VM fusion phenotypes which can be difficult to identify. While mutants such as *Alk *and *Jeb*, which do not specify founder cells [4-6,44,45] display clear fusion phenotypes which are easily identified during embryonic development, many more muscle specific genes which are expressed both in the SM and the VM, have been reported to have weak VM phenotypes when mutated, although they give severe fusion phenotypes in the SM. Examples include mutants in *rolling pebbles *[46], *antisocial *[47]*roughest *[12], *blown fuse *[48,49], *lame duck *[50,51], *loner *[52] and *kette *[49]. Our work adds *Vrp1 *the list of mutants belonging to this category.

The Vrp1 protein contains several domains, which are conserved throughout evolution (Figure 2A), [18,19]. By asking which domains of Vrp1 are required to rescue the *Vrp*^*f06715 *^mutant phenotype we have investigated the importance of the different domains of Vrp1 in *Drosophila*, and find that only the WASP-binding domain is required for muscle fusion, while the actin binding domains are dispensable. These findings are contradictory to results previously published by Kim *et. al *2007, who reported that the WH2 domains were required for rescuing the *solitary *mutant phenotype [20]. Our results indicate that the Vrp1-WASP interaction is critical in muscle fusion. However, the effects on the organization of the actin cytoskeleton, caused by Vrp1 expression in PAE cells, indicate that all conserved domains have actin cytoskeleton modulating properties, suggesting that the WH2 domains may be of importance in other contexts than myoblast fusion. Two additional proteins - Wasp and Scar - are nucleation promoting factors that act in parallel to activate the Arp2/3 complex, and mutants for the genes that encode these proteins display similar SM fusion phenotypes as the *Vrp1*^*f06715 *^mutant [26], indicating that many members of the Scar-Wasp signaling network work together to regulate myoblast fusion. We have analyzed VM fusion in additional single and double mutants for some of the components in this pathway; *kette*, *wasp*, and *arp3-wasp*, and observed that these mutants also develop a gut, suggesting that either VM fusion takes place in these mutants as in *Vrp1*^*f06715*^, or that the VM manages to develop normally despite fusion blockage. Interestingly accumulation of Duf/Kirre is observed in all examined mutants of the Scar-Wasp signaling network.

Taken together, we suggest that VM fusion is initiated in mutants of components in the Scar-Wasp signaling network, and that these molecules are involved in an increased efficiency of the fusion process.

In addition to the Arp2/3 complex, other molecular pathways are able to nucleate actin. These include proteins such as formins, Spire and Cordon-bleu. Molecules of these protein families are structurally different to the Arp2/3 complex and produce linear instead of branched actin filaments. (discussed in Campellone and Welch 2010 [53], and Aspenstöm 2010 [54]). Spire and several formins, including Diaphanous and Cappuccino, have been identified in *Drosophila*, were they have been associated with cellular processes such as vesicle transport and actin-microtubule interactions [53], but not yet with muscle development. Thus, loss of Arp2/3 function does not inhibit all actin polymerization in the cell, although the strong SM phenotypes observed in different Scar-Wasp signaling pathway mutants suggests that the Arp2/3 complex is an important actin nucleator in muscles. Our data suggests that actin polymerization by the Arp2/3 complex pathway is not required for VM fusion. Whether additional modes of actin assembly contribute to VM fusion is an interesting prospect and remains to be further investigated.

Duf/Kirre, together with Sns, is important for myoblast fusion in both the VM and the SM, as these immunoglobulin receptors facilitate attachment between FCs and FCMs, and therefore a mislocalisation of this molecule suggests that the process of fusion does not proceed in the normal fashion. We observe that Duf/Kirre protein is not downregulated in the VM of *Vrp1*^*f06715 *^mutants, possibly reflecting a stalled or inefficient fusion process. However, a recognizable embryonic gut is developed despite this phenotype, and the longitudinal muscles of *Vrp1*^*f06715 *^mutants appear morphologically wild type, suggesting that fusion defects do not affect VM development. Interestingly, we also observed a significant accumulation of Duf/Kirre protein in the SM of the analyzed *Vrp1*^*f06715 *^mutants, strengthening the hypothesis that this particular phenotype is the result of an inability of myoblasts to fuse properly. Accumulation of Duf/Kirre in the SM has previously been reported and suggested to reflect an imbalance in Duf/Rols signaling during fusion [33], a conclusion that is supported by recent study investigating Duf/Kirre signaling in myoblast fusion efficiency [55]. Our findings in the VM of *Vrp1 *mutants, together with our and others reports in the SM [20-22,26] indicate that Vrp1 and components of the Scar-Wasp signaling network are also important for fusion efficiency. Ultrastructural analysis with electron microscopy has shown that SM cell fusion is a process of many steps, including the adherence of the myoblasts to each other, the appearance of vesicles and elongated plaques on both sides of the plasma membranes, the formation of fusion pores which lead to mixing of cell content, and then an anticipated enlargement of the pores as the plasma membranes are broken down, which finally results in complete fusion of the two cells [48]. The Duf/Kirre accumulation in the mutants examined in this study may reflect an inability of fusing cells to proceed through all the above described fusion steps, resulting in an incomplete or stalled fusion event. This would still produce an obvious fusion defective phenotype in the SM, but appears to have little effect in the embryonic VM. Clearly, it remains to be investigated whether loss of Vrp1 results in later developmental defects.

As a result of our experiments investigating Vrp1 function in the VM we conclude that Vrp1 is not absolutely required for muscle cell fusion *in vivo*. This is evidenced by the fact that *Alk10-Vrp1 *double mutant FCMs originating from the VM are clearly capable of fusing with FCs of the SM. Naturally, one major difference between the fusion process in the VM and the fusion process in the SM, is that in the VM one FC fuses with only one FCM, whereas in the SM one FC per myotube fuses with up to 25 FCMs to form much larger muscle syncytia. It is possible that the many fusion events that take place in the SM require significantly more efficient actin rearrangement machinery than the few fusion events in the VM, and this would then explain why the fusion phenotypes that are caused by *Vrp1*, *scar*, *wasp *and *arp3 *disruption are more visible in the SM than in the VM. It follows that evaluation of VM developmental defects will be difficult given current markers, and that study of the VM during larval stages will provide insight. Thus, although the VM of the *Vrp1*^*f0671 *^mutant displays only minor defects at embryonic stages, the gut may be non-functional as the animal develops further. Unfortunately, at present time we are unable to test the functionality of the mutant larval gut since the *Vrp1 *mutation causes an embryonic lethal phenotype precluding an investigation of the mutant larval gut. For this, a SM specific tissue rescue would be required, something which is currently not possible. Future development of tools to allow investigation of the function of Vrp1, and indeed other molecules, in the *Drosophila *visceral muscle at later stages must now be a priority for analyzing the gut muscle specific function of Vrp1 *in vivo*.

## Methods

### Fly strains

Standard *Drosophila *husbandry procedures were followed. The following stocks were used: *w*^*1118*^, referred to as *WT *in Figures and text (Bloomington, stock number 5905), *Df(2R)ED3943 *(Bloomington, stock number 9158), *P(Tub-PBac\T)2/wg*^*Sp-1 *^(Bloomington, stock number 8285), *rp298lacZ *[31], *Vrp1*^*f06715 *^(Exelixis Collection at the Harvard Medical School [43]), *sns20*^*23*^, referred to as *sns *in Figures and text [13], *twistp-GFP-actin *[25], *UAS-LacZ*, *5053-GAL4 *[56], *kette*^*j4-48 *^[34], *Arp3*^*schwächling *^*wasp*^*3D3-035*^, referred to as *Arp3-WASP *in text [26], *wasp*^*3D3-035 *^[35], *Alk10 *[45], *Sns-GAL4 *[41]. Transgenic fly strains: *UAS-Vrp1*^*full length*^, *UAS-Vrp1*^ΔWH2^, *UAS-Vrp1*^ΔProΔWASP ^and *UAS-Vrp1*^ΔWASP ^were generated as described below.

### Crosses

*P(Tub-PBac\T)2/wg*^*Sp-1 *^flies were crossed to *Vrp1*^*f06715 *^flies to induce expression of piggyBac transposase, in order to remobilize the WH^f06715 ^element. To drive LacZ expression in the longitudinal muscles of *Vrp1*^*f06715 *^mutant as well as heterozygous controls, flies with the genotype *Vrp1-UAS:lacZ/CyOWgLacZ *were crossed to flies with the genotype *Vrp1/CyOWgLacZ; 5053-GAL4*. For studies of migration and fusion of VM cells in the SM, fly strains with the genotype *Alk*^*10*^*-Vrp1/CyOWgLacZ *were generated as well as flies with the genotype *rp298lacZ;Alk*^*10*^*-Vrp1/CyOWgLacZ *. For rescue experiments flies of the genotype *Vrp1*^*f06715*^*/CyOWgLacZ;UAS-Vrp1 transgene *(all four UAS-transgenes, Figure 8A) were crossed with flies of the genotype *Vrp1*^*f06715*^*-sns-GAL4/CyOWgLacZ*, and in the case of rescue of lethality straight winged flies were counted. For studies of actin expression in muscles a *twistp-GFP-actin*-*Vrp1*^*f06715 *^fly strain was generated via recombination.

### Generation of *Vrp1 *transgenic constructs

The *Vrp1 *cDNA clone GH25793 (Drosophila Genomics Resource Center) was used as a PCR template to generate four different myc tagged *Vrp1 *transgenic constructs; *Vrp1 full length *(2250 bp), *Vrp1 2XΔWH2 *(1830 bp), *Vrp1 ΔProΔWBD *(450 bp) and Vrp1 *ΔWBD (2140 bp)*. The primers added a *BamHI *restriction site to the 5' end of the PCR product and a *XhoI *restriction site and a myc sequence to the 3' end. Primers for *Vrp1 full length *were; 5' primer: GGA TCC GCC ATG GCT ATT CCG CCA CCC CCG GGA, 3' primer: CTC GAG CTA CAG ATC CTC TTC AGA GAT GAG TTT CTG CTC CAT ACC ATT GGT GGC CTT AAA. Primers for *Vrp1 ΔWH2 *were; 5' primer: GGA TCC GCC GCC ATG ACA ACG AAC TCA TCC GCT CAG, 3' primer: CTC GAG CTA CAG ATC CTC TTC AGA GAT GAG TTT CTG CTC CAT ACC ATT GGT GGC CTT AAA. Primers for *Vrp1 ΔProΔWBD *were; 5' primer: GGA TCC GCC ATG GCT ATT CCG CCA CCC CCG GGA, 3' primer: CTC GAG CTA CAG ATC CTC TTC AGA GAT GAG TTT CTG CTC TTG GCG CTT CAA CGT CAA GTG. Primers for *Vrp1 ΔWBD *were; 5' primer: GGA TCC GCC ATG GCT ATT CCG CCA CCC CCG GGA, 3' primer: CTC GAG CTA CAG ATC CTC TTC AGA GAT GAG TTT CTG CTC GGT CTC CAA GTC GTT GAC CAG. Standard PCR programs were used to amplify DNA fragments. PCR products were then digested with *BamHI *and *XhoI *and subcloned into the pUAST plasmid [57] and pcDNA3 (Invitrogen), and the resulting constructs were confirmed by DNA sequencing prior to injection and generation of transgenic fly strains (BestGene Inc).

### Embryo Immunostainings and *in situ *hybridization

Unless otherwise stated, embryos were collected, fixed and immunostained as described previously [58], prior to dehydration and mounting in methylsalicylate on glass slides for analysis. The following primary antibodies were used: Rabbit anti-β3 Tubulin (1:5000) [59], guinea pig anti- β3 Tubulin (1:10 000) [59], rabbit anti-βGal (1:150, Cappel), mouse anti-βGal (1:1000, Promega), mouse anti-Mef2 (1:500, gift from B. Paterson), rabbit anti-Alk (1:1000)[45], guinea pig anti-Alk (1:1000)[5], mouse anti-FasIII (1:50, Developmental Studies Hybridoma Bank), rabbit anti-Duf/Kirre (1:300)[60]. Guinea pig anti-Vrp1 was generated by injection of guinea pigs with recombinant HIS-tagged protein corresponding to residues 837-936 of Vrp1 in pETM11 [61]. The resulting guinea pig antiserum (Medprobe) was IgG-purifed on a Protein A column (Pierce) prior to use at 1:1000 for immunostaining. Fluorescent secondary antibodies employed were: goat anti-rabbit Cy3 (1:1000, Amersham), goat anti-mouse Cy3 (1:1000, Jackson), donkey anti-guinea pig Cy3 (1:200, Jackson), goat anti-rabbit Cy2 (1:1000, Amersham), goat anti-mouse Cy2 (1:1000, Amersham), donkey anti-guinea pig Cy2 (1:1000, Jackson), donkey anti-rabbit Cy5 (1:200, Jackson), donkey anti-mouse Cy5 (1:200, Jackson), donkey anti-guinea pig Cy5 (1:400, Jackson). For *in situ *hybridization a digoxigenin-labelled RNA probe was made using cDNA encoding *Vrp1 *and a DIG RNA labelling kit (Roche). *In situ *hybridization of whole-mount wild type *Drosophila *embryos was carried out as described [62].

### Cell line experiments

Porcine aortic endothelial (PAE ) cells were cultured in Ham's F12 medium, Supplemented with 10% FBS and penicillin/streptomycin at 37°C in an atmosphere of 5% CO2. For immunstaining experiments, the cells were seeded on coverslips and transiently transfected by Lipofectamine (Invitrogen Life Technologies) employing the protocol provided by the manufacturer. Twenty hours post-transfection, the cells were fixed in 3% paraformaldehyde in phosphate buffered saline (PBS) for 20 minutes at 37°C and washed with PBS. The cells were thereafter permeabilized in 0.2% Triton X-100 in PBS for 5 minutes, washed again in PBS and incubated in 5% FBS in PBS for 30 minutes at room temperature. To visualize filamentous actin, cells were incubated with tetramethyl rhodamine isothiocyanate (TRITC)-conjugated phalloidin (Sigma) diluted in 5% FBS in PBS for 30 minutes at room temperature. The coverslips were washed in PBS and mounted on object slides by the use of Fluoromount-G (Southern Biotechnology Associates). Cells were photographed by a Hamamatsu ORCA CCD digital camera employing the QED Imaging System software using a Zeiss Axioplan2 microscope. Thick bundles, actin dots and stress fibers were quantified manually in microscope by calculating the percentage of transfected PAE cells displaying these structures or cells displaying extensive loss of stress fibers (see legends to Figure 8). All samples were analyzed blind.

### List of abbreviations

Arp2/3: Actin-related protein 2 and 3; ALK: Anaplastic lymphoma kinase; lacZ: Beta galactosidase; Duf/Kirre: Dumbfounded/Kin of Irre; FasIII: Fasciclin III; FCs: founder cells; FCMs: fusion competent myoblasts; PAE: porcine aortic endothelial; RTK: receptor tyrosine kinase; Scar: suppressor of cAMP receptor; SM: somatic mesoderm; SNS: Sticks and Stones; Vrp1: Verprolin; VCA: Verprolin-homology, cofilin-homology, and highly acidic; VM: visceral mesoderm; Wip: Wasp interacting protein; Wasp: Wiskott-Aldrich syndrome protein

## Authors' contributions

TE and RHP designed the study, TE carried out the genetic and molecular characterization, PA carried out the PAE cell experiments and GV performed the *in situ *hybridization analysis TE, PA and RHP wrote the manuscript. All authors read and approved the final manuscript.

## Supplementary Material

Additional file 1 **Supplemental Figure 1; Comparison of SM phenotypes between the *Vrp1*^*f06715 *^and *WIP*^*D30*^**. Comparison of SM phenotypes between the *Vrp1*^*f06715 *^and *WIP*^*D30 *^mutants reveals a similar degree of myoblast fusion defects in both mutants. Somatic embryonic muscles are stained with β3-Tubulin antibodies. (A) *Wild type *embryo, (B) *Vrp1*^*f06715*^, (C) *D-WIP*^*D30*^, (D) *Vrp1*^*f06715*^*/D-WIP*^*D30 *^transheterozygotes. Click here for file

Additional file 2 **Supplemental Figure 2; Rescue experiments of *Vrp1*^*f06715 *^mutant embryos with different *Vrp1 *constructs, and description of mutant phenotypes observed in PAE cells upon expression of the different Vrp1 constructs**. Rescue of the *Vrp1*^*f06715 *^embryonic mutant phenotype performed with different Vrp1 constructs as described in Figure 8. *UAS-Vrp1*^*full length *^and *UAS-Vrp1*^*Δ2xWH2 *^are both able to fully rescue the SM fusion phenotype of the *Vrp1*^*f06715 *^mutant when expressed with the Sns-Gal4 driver, while *UAS-Vrp1*^*ΔWBD *^and *UAS-Vrp1*^*ΔProΔWBD *^are not. A representative embryo from each cross is shown. Unfused cells are indicated by arrows. (A) *Vrp1*^*f06715*^*Sns > > Vrp1*^*full length *^(B) *Vrp1*^*f06715*^*Sns > > Vrp1*^*Δ2xWH2*^. (C) *Vrp1*^f06715^*;Sns > > UAS-Vrp1*^*ΔProΔWBD*^. (D) *Vrp1*^*f06715*^*;Sns > > UAS-Vrp1*^*ΔWBD*^. (E) The white arrow indicates normal stress fibers (SF). Non transfected PAE cells contain numerous stress fibers in contrast to cells that ectopically express full length Vrp1. The Vrp1-expressing cells undergo a very characteristic reorganization of the actin filament system; the cells appear almost empty of the bulk filamentous actin, apart from few and thick bundles of actin filaments and a formation of focal points of actin, so called actin dots. Red arrows indicate the presence of thick bundles and actin dots, as well as stress fiber loss (SF loss). Click here for file

## References

[B1] Klapper R Stute C Schomaker O Strasser T Janning W Renkawitz-Pohl R Holz A The formation of syncytia within the visceral musculature of the Drosophila midgut is dependent on duf, sns and mbc Mech Dev 2002 110 85 96 10.1016/S0925-4773(01)00567-6 11744371

[B2] Georgias C Wasser M Hinz U A basic-helix-loop-helix protein expressed in precursors of Drosophila longitudinal visceral muscles Mech Dev 1997 69 115 24 10.1016/S0925-4773(97)00169-X 9486535

[B3] Martin BS Ruiz-Gomez M Landgraf M Bate M A distinct set of founders and fusion-competent myoblasts make visceral muscles in the Drosophila embryo Development 2001 128 3331 8 11546749 10.1242/dev.128.17.3331

[B4] Lee HH Norris A Weiss JB Frasch M Jelly belly protein activates the receptor tyrosine kinase Alk to specify visceral muscle pioneers Nature 2003 425 507 12 10.1038/nature01916 14523446

[B5] Englund C Loren CE Grabbe C Varshney GK Deleuil F Hallberg B Palmer RH Jeb signals through the Alk receptor tyrosine kinase to drive visceral muscle fusion Nature 2003 425 512 6 10.1038/nature01950 14523447

[B6] Stute C Schimmelpfeng K Renkawitz-Pohl R RH Palmer Holz A Myoblast determination in the somatic and visceral mesoderm depends on Notch signalling as well as on milliways(mili(Alk)) as receptor for Jeb signalling Development 2004 131 743 54 10.1242/dev.00972 14757637

[B7] Lee H-H Zaffran S Frasch M Development of the Larval Visceral Musculature 2006 1 Austin: Landes Bioscience

[B8] Richardson BE Nowak SJ Baylies MK Myoblast fusion in fly and vertebrates: new genes, new processes and new perspectives Traffic 2008 9 1050 9 10.1111/j.1600-0854.2008.00756.x 18435820 PMC2677912

[B9] Taylor MV Muscle development: molecules of myoblast fusion Curr Biol 2000 10 R646 8 10.1016/S0960-9822(00)00664-3 10996092

[B10] Richardson BE Beckett K Baylies MK Live imaging of Drosophila myoblast fusion Methods Mol Biol 2008 475 263 74 full_text 18979249 10.1007/978-1-59745-250-2_15 PMC2883172

[B11] Abmayr SM Zhuang S Geisbrecht ER Myoblast fusion in Drosophila Methods Mol Biol 2008 475 75 97 full_text 18979239 10.1007/978-1-59745-250-2_5

[B12] Strunkelnberg M Bonengel B Moda LM Hertenstein A de Couet HG Ramos RG Fischbach KF rst and its paralogue kirre act redundantly during embryonic muscle development in Drosophila Development 2001 128 4229 39 11684659 10.1242/dev.128.21.4229

[B13] Ruiz-Gomez M Coutts N Price A Taylor MV Bate M Drosophila dumbfounded: a myoblast attractant essential for fusion Cell 2000 102 189 98 10.1016/S0092-8674(00)00024-6 10943839

[B14] Dworak HA Sink H Myoblast fusion in Drosophila Bioessays 2002 24 591 601 10.1002/bies.10115 12111720

[B15] Galletta BJ Chakravarti M Banerjee R Abmayr SM SNS: Adhesive properties, localization requirements and ectodomain dependence in S2 cells and embryonic myoblasts Mech Dev 2004 121 1455 68 10.1016/j.mod.2004.08.001 15511638

[B16] Takenawa T Suetsugu S The WASP-WAVE protein network: connecting the membrane to the cytoskeleton Nat Rev Mol Cell Biol 2007 8 37 48 10.1038/nrm2069 17183359

[B17] Miki H Takenawa T Regulation of actin dynamics by WASP family proteins J Biochem 2003 134 309 13 10.1093/jb/mvg146 14561714

[B18] Aspenstrom P The verprolin family of proteins: regulators of cell morphogenesis and endocytosis FEBS Lett 2005 579 5253 9 10.1016/j.febslet.2005.08.053 16182290

[B19] Paunola E Mattila PK Lappalainen P WH2 domain: a small, versatile adapter for actin monomers FEBS Lett 2002 513 92 7 10.1016/S0014-5793(01)03242-2 11911886

[B20] Kim S Shilagardi K Zhang S Hong SN Sens KL Bo J Gonzalez GA Chen EH A critical function for the actin cytoskeleton in targeted exocytosis of prefusion vesicles during myoblast fusion Dev Cell 2007 12 571 86 10.1016/j.devcel.2007.02.019 17419995

[B21] Massarwa R Carmon S Shilo BZ Schejter ED WIP/WASp-based actin-polymerization machinery is essential for myoblast fusion in Drosophila Dev Cell 2007 12 557 69 10.1016/j.devcel.2007.01.016 17419994

[B22] Estrada B Choe SE Gisselbrecht SS Michaud S Raj L Busser BW Halfon MS Church GM Michelson AM An integrated strategy for analyzing the unique developmental programs of different myoblast subtypes PLoS Genet 2006 2 e16 10.1371/journal.pgen.0020016 16482229 PMC1366495

[B23] Smith LG Li R Actin polymerization: riding the wave Curr Biol 2004 14 R109 11 14986640

[B24] Rushton E Drysdale R Abmayr SM Michelson AM Bate M Mutations in a novel gene, city myoblast, provide evidence in support of the founder cell hypothesis for Drosophila muscle development Development 1995 121 1979 88 7635046 10.1242/dev.121.7.1979

[B25] Richardson BE Beckett K Nowak SJ Baylies MK SCAR/WAVE and Arp2/3 are crucial for cytoskeletal remodeling at the site of myoblast fusion Development 2007 134 4357 67 10.1242/dev.010678 18003739 PMC2880884

[B26] Berger S Schafer G Kesper DA Holz A Eriksson T Palmer RH Beck L Klambt C Renkawitz-Pohl R Onel SF WASP and SCAR have distinct roles in activating the Arp2/3 complex during myoblast fusion J Cell Sci 2008 121 1303 13 10.1242/jcs.022269 18388318

[B27] Schroter RH Lier S Holz A Bogdan S Klambt C Beck L Renkawitz-Pohl R kette and blown fuse interact genetically during the second fusion step of myogenesis in Drosophila Development 2004 131 4501 9 10.1242/dev.01309 15342475

[B28] Luo L Liao YJ Jan LY Jan YN Distinct morphogenetic functions of similar small GTPases: Drosophila Drac1 is involved in axonal outgrowth and myoblast fusion Genes Dev 1994 8 1787 802 10.1101/gad.8.15.1787 7958857

[B29] Hakeda-Suzuki S Ng S Tzu J Dietzl G Sun Y Harms M Nardine T Luo L Dickson BJ Rac function and regulation during Drosophila development Nature 2002 416 438 42 10.1038/416438a 11919634

[B30] Estrada B Michelson AM A genomic approach to myoblast fusion in Drosophila Methods Mol Biol 2008 475 299 314 full_text 18979251 10.1007/978-1-59745-250-2_17 PMC3190861

[B31] Nose A Isshiki T Takeichi M Regional specification of muscle progenitors in Drosophila: the role of the msh homeobox gene Development 1998 125 215 23 9486795 10.1242/dev.125.2.215

[B32] Bour BA Chakravarti M West JM Abmayr SM Drosophila SNS, a member of the immunoglobulin superfamily that is essential for myoblast fusion Genes Dev 2000 14 1498 511 10859168 PMC316690

[B33] Menon SD Osman Z Chenchill K Chia W A positive feedback loop between Dumbfounded and Rolling pebbles leads to myotube enlargement in Drosophila J Cell Biol 2005 169 909 20 10.1083/jcb.200501126 15955848 PMC2171639

[B34] Hummel T Leifker K Klambt C The Drosophila HEM-2/NAP1 homolog KETTE controls axonal pathfinding and cytoskeletal organization Genes Dev 2000 14 863 73 10766742 PMC316499

[B35] Schafer G Weber S Holz A Bogdan S Schumacher S Muller A Renkawitz-Pohl R Onel SF The Wiskott-Aldrich syndrome protein (WASP) is essential for myoblast fusion in Drosophila Dev Biol 2007 304 664 74 10.1016/j.ydbio.2007.01.015 17306790

[B36] Chou HC Anton IM Holt MR Curcio C Lanzardo S Worth A Burns S Thrasher AJ Jones GE Calle Y WIP regulates the stability and localization of WASP to podosomes in migrating dendritic cells Curr Biol 2006 16 2337 44 10.1016/j.cub.2006.10.037 17141616 PMC1885947

[B37] Theriot JA Mitchison TJ Actin microfilament dynamics in locomoting cells Nature 1991 352 126 31 10.1038/352126a0 2067574

[B38] Sawa M Suetsugu S Sugimoto A Miki H Yamamoto M Takenawa T Essential role of the C. elegans Arp2/3 complex in cell migration during ventral enclosure J Cell Sci 2003 116 1505 18 10.1242/jcs.00362 12640035

[B39] Suetsugu S Yamazaki D Kurisu S Takenawa T Differential roles of WAVE1 and WAVE2 in dorsal and peripheral ruffle formation for fibroblast cell migration Dev Cell 2003 5 595 609 10.1016/S1534-5807(03)00297-1 14536061

[B40] Williamson MP The structure and function of proline-rich regions in proteins Biochem J 1994 297 Pt 2 249 60 8297327 10.1042/bj2970249 PMC1137821

[B41] Stute C Dörthe K Holz A Buttgerei D Renkawitz-Pohl R Establishment of cell type specific Gal4-driver lines for the mesoderm of Drosophila Dros Inf Serv 2006 89 111 115

[B42] Aspenstrom P The mammalian verprolin homologue WIRE participates in receptor-mediated endocytosis and regulation of the actin filament system by distinct mechanisms Exp Cell Res 2004 298 485 98 10.1016/j.yexcr.2004.04.050 15265696

[B43] Thibault ST Singer MA Miyazaki WY Milash B Dompe NA Singh CM Buchholz R Demsky M Fawcett R Francis-Lang HL A complementary transposon tool kit for Drosophila melanogaster using P and piggyBac Nat Genet 2004 36 283 7 10.1038/ng1314 14981521

[B44] Weiss JB Suyama KL Lee HH Scott MP Jelly belly: a Drosophila LDL receptor repeat-containing signal required for mesoderm migration and differentiation Cell 2001 107 387 98 10.1016/S0092-8674(01)00540-2 11701128

[B45] Loren CE Englund C Grabbe C Hallberg B Hunter T Palmer RH A crucial role for the Anaplastic lymphoma kinase receptor tyrosine kinase in gut development in Drosophila melanogaster EMBO Rep 2003 4 781 6 10.1038/sj.embor.embor897 12855999 PMC1326337

[B46] Menon SD Chia W Drosophila rolling pebbles: a multidomain protein required for myoblast fusion that recruits D-Titin in response to the myoblast attractant Dumbfounded Dev Cell 2001 1 691 703 10.1016/S1534-5807(01)00075-2 11709189

[B47] Chen EH Olson EN Antisocial, an intracellular adaptor protein, is required for myoblast fusion in Drosophila Dev Cell 2001 1 705 15 10.1016/S1534-5807(01)00084-3 11709190

[B48] Doberstein SK Fetter RD Mehta AY Goodman CS Genetic analysis of myoblast fusion: blown fuse is required for progression beyond the prefusion complex J Cell Biol 1997 136 1249 61 10.1083/jcb.136.6.1249 9087441 PMC2132517

[B49] Schroter RH Buttgereit D Beck L Holz A Renkawitz-Pohl R Blown fuse regulates stretching and outgrowth but not myoblast fusion of the circular visceral muscles in Drosophila Differentiation 2006 74 608 21 10.1111/j.1432-0436.2006.00080.x 17177857

[B50] Duan H Skeath JB Nguyen HT Drosophila Lame duck, a novel member of the Gli superfamily, acts as a key regulator of myogenesis by controlling fusion-competent myoblast development Development 2001 128 4489 500 11714674 10.1242/dev.128.22.4489

[B51] Ruiz-Gomez M Coutts N Suster ML Landgraf M Bate M myoblasts incompetent encodes a zinc finger transcription factor required to specify fusion-competent myoblasts in Drosophila Development 2002 129 133 41 11782407 10.1242/dev.129.1.133

[B52] Chen EH Pryce BA Tzeng JA Gonzalez GA Olson EN Control of myoblast fusion by a guanine nucleotide exchange factor, loner, and its effector ARF6 Cell 2003 114 751 62 10.1016/S0092-8674(03)00720-7 14505574

[B53] Campellone KG Welch MD A nucleator arms race: cellular control of actin assembly Nat Rev Mol Cell Biol 2010 11 237 51 10.1038/nrm2867 20237478 PMC2929822

[B54] Aspenstrom P Formin-binding proteins: modulators of formin-dependent actin polymerization Biochim Biophys Acta 2010 1803 174 82 10.1016/j.bbamcr.2009.06.002 19589360

[B55] Bulchand S Menon SD George SE Chia W The intracellular domain of Dumbfounded affects myoblast fusion efficiency and interacts with Rolling pebbles and Loner PLoS One 5 e9374 10.1371/journal.pone.0009374 20186342 PMC2826419

[B56] Ritzenthaler S Suzuki E Chiba A Postsynaptic filopodia in muscle cells interact with innervating motoneuron axons Nat Neurosci 2000 3 1012 7 10.1038/79833 11017174

[B57] Brand AH Perrimon N Targeted gene expression as a means of altering cell fates and generating dominant phenotypes Development 1993 118 401 15 8223268 10.1242/dev.118.2.401

[B58] Patel NH Imaging neuronal subsets and other cell types in whole-mount Drosophila embryos and larvae using antibody probes Methods Cell Biol 1994 44 445 87 full_text 7707967 10.1016/s0091-679x(08)60927-9

[B59] Leiss D Hinz U Gasch A Mertz R Renkawitz-Pohl R Beta 3 tubulin expression characterizes the differentiating mesodermal germ layer during Drosophila embryogenesis Development 1988 104 525 31 3077351 10.1242/dev.104.4.525

[B60] Kreiskother N Reichert N Buttgereit D Hertenstein A Fischbach KF Renkawitz-Pohl R Drosophila Rolling pebbles colocalises and putatively interacts with alpha-Actinin and the Sls isoform Zormin in the Z-discs of the sarcomere and with Dumbfounded/Kirre, alpha-Actinin and Zormin in the terminal Z-discs J Muscle Res Cell Motil 2006 27 93 106 10.1007/s10974-006-9060-y 16699917

[B61] Dummler A Lawrence AM de Marco A Simplified screening for the detection of soluble fusion constructs expressed in E. coli using a modular set of vectors Microb Cell Fact 2005 4 34 10.1186/1475-2859-4-34 16351710 PMC1326211

[B62] Kopczynski CC Davis GW Goodman CS A neural tetraspanin, encoded by late bloomer, that facilitates synapse formation Science 1996 271 1867 70 10.1126/science.271.5257.1867 8596956

