# Supplementary figures and images for: Characterisation of the role of Vrp1 in cell fusion during the development of visceral muscle of Drosophila melanogaster
Source: BMC Dev Biol. 2010 Aug 11;10:86. doi: 10.1186/1471-213X-10-86 (PMC2931478; doi:10.1186/1471-213X-10-86)

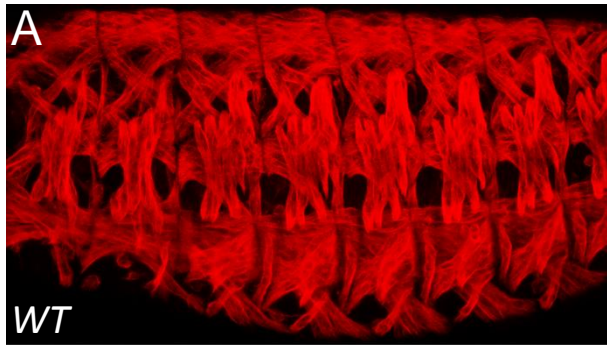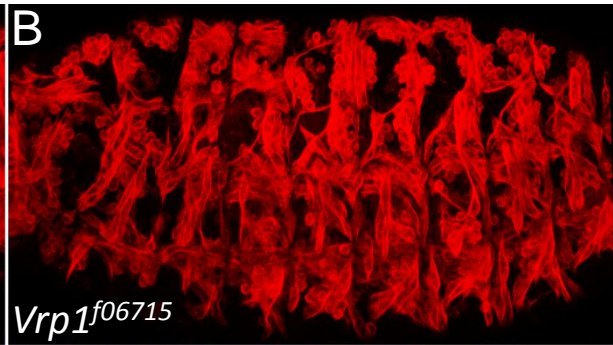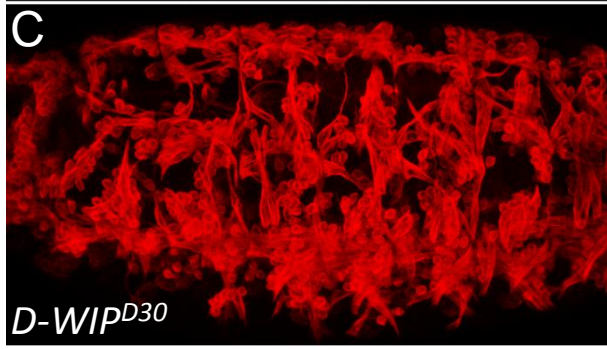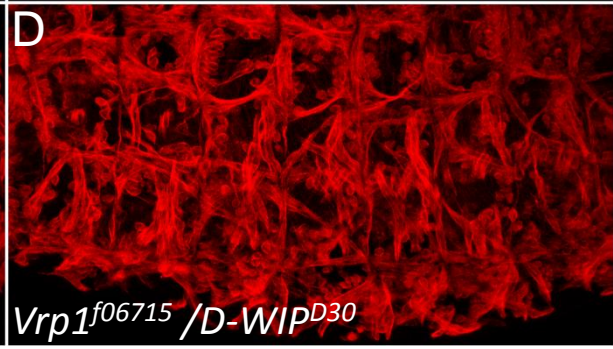

Supplement: Additional file 1 — Supplemental Figure 1; Comparison of SM phenotypes between the Vrp1f06715 and WIPD30. Comparison of SM phenotypes between the Vrp1f06715 and WIPD30 mutants reveals a similar degree of myoblast fusion defects in both mutants. Somatic embryonic muscles are stained with β3-Tubulin antibodies. (A) Wild type embryo, (B) Vrp1f06715, (C) D-WIPD30, (D) Vrp1f06715/D-WIPD30 transheterozygotes. [file 1471-213X-10-86-S1.PDF]

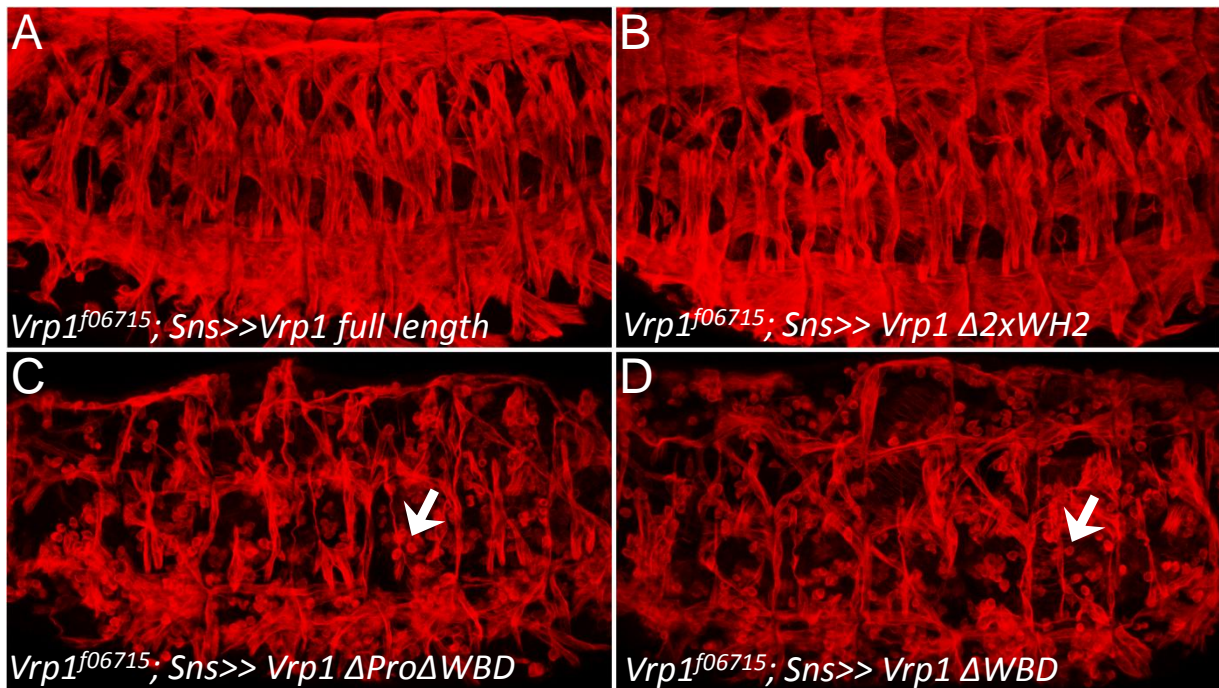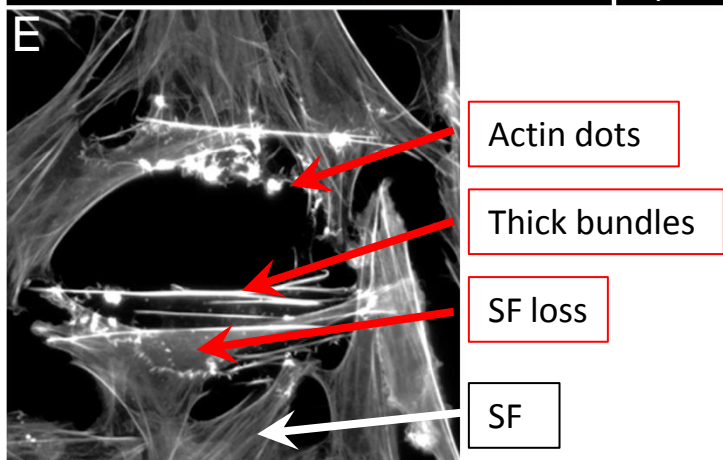

Supplement: Additional file 2 — Supplemental Figure 2; Rescue experiments of Vrp1f06715 mutant embryos with different Vrp1 constructs, and description of mutant phenotypes observed in PAE cells upon expression of the different Vrp1 constructs. Rescue of the Vrp1f06715 embryonic mutant phenotype performed with different Vrp1 constructs as described in Figure 8. UAS-Vrp1full length and UAS-Vrp1Δ2xWH2 are both able to fully rescue the SM fusion phenotype of the Vrp1f06715 mutant when expressed with the Sns-Gal4 driver, while UAS-Vrp1ΔWBD and UAS-Vrp1ΔProΔWBD are not. A representative embryo from each cross is shown. Unfused cells are indicated by arrows. (A) Vrp1f06715Sns > > Vrp1full length (B) Vrp1f06715Sns > > Vrp1Δ2xWH2. (C) Vrp1f06715;Sns > > UAS-Vrp1ΔProΔWBD. (D) Vrp1f06715;Sns > > UAS-Vrp1ΔWBD. (E) The white arrow indicates normal stress fibers (SF). Non transfected PAE cells contain numerous stress fibers in contrast to cells that ectopically express full length Vrp1. The Vrp1-expressing cells undergo a very characteristic reorganization of the actin filament system; the cells appear almost empty of the bulk filamentous actin, apart from few and thick bundles of actin filaments and a formation of focal points of actin, so called actin dots. Red arrows indicate the presence of thick bundles and actin dots, as well as stress fiber loss (SF loss). [file 1471-213X-10-86-S2.PDF]
